# From Molecular to Macroscopic: Dual-Pathway Regulation of Carrot Whole Flour on the Gluten-Starch System

**DOI:** 10.3390/foods14111964

**Published:** 2025-05-31

**Authors:** Han Wang, Xiaoxuan Tian, Ruoyu Zhang, Huijing Li

**Affiliations:** College of Food Science and Technology, Hebei Agricultural University, Baoding 071001, China; wh19972024@163.com (H.W.); 18233219394@163.com (X.T.); zryzry2000@163.com (R.Z.)

**Keywords:** carrot flour, wheat flour, steamed cake, gluten, starch

## Abstract

Carrots are gaining attention due to their health effects, high yield, low cost, and bright color in food processing. This study analyzed the impact of carrot whole flour (CWF) on steamed cake quality. The effects of CWF and its active ingredients, carrot dietary fiber (CDF) and carrot polyphenols (CPs), on gluten and starch properties were studied. Results showed that steamed cake quality was better at a 12% additional dose. CPs caused gluten to form more hydrogen bonds, increasing the specific volume. CDF weakened the gluten structure by reducing disulfide bonds, decreasing the hardness. Both CDF and CPs disrupted the starch structure by decreasing the short-range order, causing a reduction in springiness and cohesiveness. CDF and CPs increased starch crystallinity, which also contributed to decreasing springiness. This study systematically evaluated the effect of CWF on the steamed cake from the microstructure level to macroscopic quality. Wheat-vegetable blend flour is a key path for nutritional upgrading of traditional staple foods and an essential direction for functional wheat products.

## 1. Introduction

As a traditional fermented food in China, steamed cake is made by fermenting batter and then steaming it, giving it a soft, elastic texture and a continuous, spongy internal network [1]. It is widely consumed and very popular in China. Compared to traditional bakery products, more nutrients are retained in steamed cake because of the comparatively low steaming temperature [2]. The credo “Make food your medicine, make medicine your food” invoked by Hippocrates nearly 2500 years ago has recently rekindled scientific interest, sparking a passion for studying bioactive compounds and functional food [3]. Studies had revealed that adding plant-based ingredients such as oyster mushroom flour [4] and grape pomace flour [5] can enhance the nutritional value of wheat products. Carrots are one of the root vegetables that are widely consumed around the world. Furthermore, carrots serve as an effective food source due to their substantial contribution to diverse nutraceuticals, such as carotenoids, dietary fiber, and phenolics [6]. These natural products are certainly of the essence in averting various illnesses, including heart disease, eye retinal conditions, and cancer [7].

There are many different techniques for drying fruits and vegetables, and freeze-drying is recognized as the method that retains the most nutrients [8]. However, freeze-drying is costly [9]. The production cost of freeze-drying is 4 times that of spray drying and 8 times that of hot air drying, and the energy consumption is 4 to 10 times that of hot air drying, which limits the use of this technology to high-value products. The food industry has shown a keen interest in microwave drying, attracted by its ability to provide volumetric heating, enhance drying speed, improve the quality of products, and partially eliminate water that has adhered [10]. Microwave drying could dry various fruits and vegetables, such as orange-fleshed sweet potato chips [11], apples [12], and pleurotus eryngii [13]. Continuous application of microwave radiation has the disadvantages of uneven heating and quality degradation. Most often, microwave energy is applied intermittently. The advantages of intermittent microwave drying include short drying times; low energy consumption; high drying capacity; and better preservation of colors, flavors, and bioactive compounds [14].

Protein and starch are the most important ingredients in wheat flour. Wheat protein is mainly divided into albumin, globulin, glutenin, and gliadin. Glutenin and gliadin jointly stabilize the gluten matrix, making up roughly 80% of the gluten protein’s dry weight. Gluten is essential in determining the viscoelasticity, extensibility, and plasticity, and it greatly influences the overall quality of wheat-based products [15]. Batter is a system consisting of a gluten matrix, a protein network, and starch granules [16]. The starch particles are located within the gluten network to help keep the gluten matrix stable [17]. When carrot whole flour is mixed with wheat flour, the dietary fiber and polyphenols from the carrot flour interact with the wheat starch and protein during the process of forming the steamed cake, greatly influencing the final quality of the steamed cake.

At present, carrot whole flour is a type of functional food ingredient used to realize diversified applications in foods. There were some reports regarding wheat-carrot products focused on dough characteristics, physicochemical properties, and nutritional value [18,19,20]. Nevertheless, the quantitative correlation studies between the structure (covalent bonds, noncovalent bonds, short-range ordered, and long-range ordered) of the gluten-starch composite matrix in the processing system of CWF steamed cake and the key indicators (specific volume, texture) are limited. Therefore, the quality in texture, specific volume, and sensory properties of CWF steamed cake were investigated. The gluten structure (free sulfhydryl, disulfide bond, intermolecular interaction force, and secondary structure) were evaluated. The starch structure (short-range ordered, long-range ordered) and pasting properties were also determined. We explored the relationship between CWF-induced changes in gluten-starch structure and the CWF steamed cake quality. This study provided the foundation for the application of fruit and vegetable powders in wheat-based products.

## 2. Materials and Methods

### 2.1. Materials

Wheat flour (WF) was procured from Wudeli Co., Ltd. (Handan, China), and carrots were sourced from the retail market (Baoding, China). The carrot slices were placed in a microwave oven (40 °C, 500 W, G80F23CN3XLN-R6K(R9), Guangdong Galanz Group Co., Ltd., Foshan, China) and heated for 20 s with 60 s intervals, and the microwave drying time was 2 h. Afterward, microwave-dried carrot slices were ground for 10 s with 60 s intervals by an experimental pulverizer (25,000 rpm, AF-04A, Yongkang Red Sun Electromechanical Co., Wenzhou, China) and sieved through an 80-mesh screen to produce carrot whole flour (CWF).

### 2.2. Determination of Chemical Compositions

Crude protein (AOAC 978.04), moisture AOAC (2000), fat (AOAC 920.85), and ash (AOAC 923.03) were assessed based on standard analytical methods. Dietary fiber content was measured following the enzymatic method, aligning with AOAC 994.13. Total polyphenols (TPs) were quantified via the Folin–Ciocalteu method. The analysis of carotenoid content adhered to the methodology outlined by Landim Parente et al. [21].

### 2.3. Preparation of Steamed Cake

The steamed cake batter was prepared by mixing 300 g wheat flour, 4.5 g yeast, 1.5 g baking powder, and 270 g water. Different levels of carrot flour (0%, 4%, 8%, 12%, and 16% w/w based on wheat flour) were added. All the ingredients were blended with water to obtain a homogeneous batter (DKM201, Guangdong Shunde Diyi Utility Electric Technology Co., Foshan, China) for 6 min at medium speed, which was then fermented (60 min, 35 °C, 80% RH; DIYATE MXF-A, Shandong Meiying Food Equipment Co. Ltd., Jinan, China). Then, every 100 g of batter was poured into a mold (4 inches, 11.5 cm diameter × 4.5 cm height), followed by fermenting for 20 min again, and subsequently was steamed by an electric steamer (Z06YA3B-G2, Shandong Duoxing Electric Appliance Co., Zibo, China) for 30 min. The number of samples per batch was 15.

### 2.4. Steamed Cake Characteristics

#### 2.4.1. Specific Volume

The specific volume of steamed cake was measured via rapeseed displacement volume (mL) with subsequent normalization to sample weight (g).

#### 2.4.2. Texture Analysis

The texture characteristics of the steamed cake were evaluated using a texture analyzer (TMS-PRO, Ying Sheng Hengtai Technology Co., Ltd., Beijing, China) fitted with a 25 mm diameter cylindrical probe (P/25). Central cake sections with uniform 25 mm thickness were prepared and positioned on the testing platform. The conditions were as follows: a compression ratio of 50%, a testing speed of 1 mm/s, and an initial force of 5 g [22].

#### 2.4.3. Color

The steamed cake (top side) color was measured by a chromameter (NR60CP, Shenzhen Sanenchi Technology Company) using the CIE-L*a*b* parameters. L* values represented lightness on a scale from 0 (absolute black) to 100 (pure white), a* values indicated chromaticity along the red–green axis (positive values = redness; negative values = greenness), b* values represented chromaticity along the yellow–blue axis (positive values = yellowness; negative values = blueness). Total color difference (ΔE) was calculated using the following equation:

(1) $$\Delta E=\sqrt{{\left(L-L_{O}\right)}^{2}+{\left(A-A_{0}\right)}^{2}+{\left(B-B_{0}\right)}^{2}}$$

#### 2.4.4. Sensory Evaluation

Sensory analysis was conducted by 10 panelists (20–30 years) adapting the method by Zhu and He et al. [23,24]. Panelists assessed the textural characteristics and appearance of the steamed cake using a rating scale. Parameters included color, structure, odor, taste, and mouthfeel. The 100-point scoring system comprised the specific volume (20%), color (15%), structure (20%), odor (15%), taste (10%), and mouthfeel (20%). The sensory evaluation standard of the steamed cake was shown in Supplementary Material Table S1.

### 2.5. Preparation of Samples

#### 2.5.1. Preparation of Dietary Fiber and Polyphenols

Carrot dietary fiber (CDF) was extracted according to the method described by Dong et al. [25]. The extraction of carrot polyphenols (CPs) was performed following the method described by Dong et al. [26]. In order to exclude the effect of other components in CWF, equal levels of CDF and CPs were used for adding; the amount of CDF and CPs added to the flour were 2.24 g/100 g and 60 μL/100 g, respectively.

#### 2.5.2. Preparation of Gluten and Starch

Wheat starch and gluten were separated using the handwashing method. CWF and its active ingredients (CDF and CPs) were added to wheat flour (100 g) and mixed with distilled water (50 mL) to form the dough, which was washed with water to separate the gluten and starch. The starch-water mixture was centrifuged (1760× *g*, 15 min) [27]. Starch and wet gluten were vacuum freeze-dried (BenchTop Pro, SP Scientific, Warminster, PA, USA) and milled by an experimental pulverizer (MFJ-W153, Beijing Liren Science and Technology Co., Beijing, China). The starch and gluten were passed through an 80-mesh sieve and refrigerated at 4 °C.

### 2.6. Gluten Properties

#### 2.6.1. Free Sulfhydryl and Disulfide Bonds Analysis

Free sulfhydryl and total sulfhydryl contents were measured according to the method of Tian et al. [28]. The gluten powder (100 mg) was mixed with 10 mL of Tris-Gly-8M urea and stirred magnetically at 400 rpm for 10 min after centrifugation for 10 min at 3500× *g*, and the supernatant was analyzed.

For free sulfhydryl, Ellman’s reagent (0.1 mL) was added to 4 mL of supernatant and kept in the dark for 20 min, and the absorbance value was determined at 412 nm.

For total sulfhydryl, a mixture of 0.4 mL of supernatant, 1.6 mL of Tris-Gly-8M urea, and 0.04 mL of β-mercaptoethanol was prepared and kept in the dark for 1 h. The mixture was treated with 4 mL of 12% (*w*/*v*) trichloroacetic acid (TCA) and incubated for another hour in the dark. Afterward, the mixture was centrifuged for 10 min at 3500× *g*. The precipitate was collected and washed twice with 5 mL of 12% TCA to remove residual β-mercaptoethanol. The final precipitate was dissolved in 4 mL of Tris-Gly-8M urea, mixed with 0.1 mL Ellman’s reagent, and kept in the dark for 20 min, and the absorbance value was determined at 412 nm.

#### 2.6.2. Intermolecular Interactions Analysis

To investigate specific molecular interactions, samples were treated with four different extraction buffers (all prepared in 0.05 M phosphate buffer, pH 7.0): 0.05 M NaCl (S1), 0.6 M NaCl (S2), 0.6 M NaCl + 1.5 M urea (S3), and 0.6 M NaCl + 8 M urea (S4). For each treatment, 15 mg samples were homogenized with 1 mL of the respective buffer, followed by centrifugation (10,000× *g*, 20 min) and subsequent stirring for 1 h. Supernatant protein content was quantified using the Bradford reagent (Brilliant Blue G-250) [29].

#### 2.6.3. Secondary Structure Analysis

The secondary structure of freeze-dried gluten samples was characterized by Fourier-transform infrared spectroscopy (FTIR) (Nexus 670, Thermo Electron Corporation, Waltham, MA, USA) following the methodology of Fu et al. [30]. Spectra were acquired in the region of 400–4000 cm^−1^, featuring 64 scans and 4 cm^−1^ resolution.

### 2.7. Starch Properties

#### 2.7.1. Pasting Properties

The pasting properties of starch samples were analyzed using a Rapid Visco Analyzer (RVA-4800, Perten Instrument, Hägersten, Sweden) following the method of Cai et al. [31]. The testing protocol consisted of (1) initial premixing at 960 rpm for 10 s, (2) maintaining at 160 rpm for 1 min at 50 °C, (3) heating to 95 °C over 7.5 min, and (4) holding at 95 °C for 5 min.

#### 2.7.2. Short-Range Order

Short-range order was determined according to Section 2.6.3.

#### 2.7.3. Starch Crystallinity

The starch samples were scanned by an X-ray diffractometer (Bruker D2PHASER, Karlsruhe, Germany). The conditions were set as follows: Cu Kα radiation at 30 kV/10 mA scanning from 5° to 50° (2θ) at 2°/min with 0.02° step size [32]. The diffraction patterns were processed using Origin 2023b software to calculate relative crystallinity, defined as the ratio of crystalline peak areas to the total diffracted area.

### 2.8. Statistical Analysis

Data were processed and analyzed by one-way ANOVA using SPSS 27.0 statistical software, and the Duncan test was chosen to compare the differences between multiple groups of experimental data (*p* < 0.05). Data visualization was conducted in Origin 2023b.

## 3. Results

### 3.1. Chemical Compositions

The chemical composition of wheat flour and carrot flour is summarized in Table 1.

### 3.2. Steamed Cake Characteristics

#### 3.2.1. Volumetric Properties

**Figure 1:**
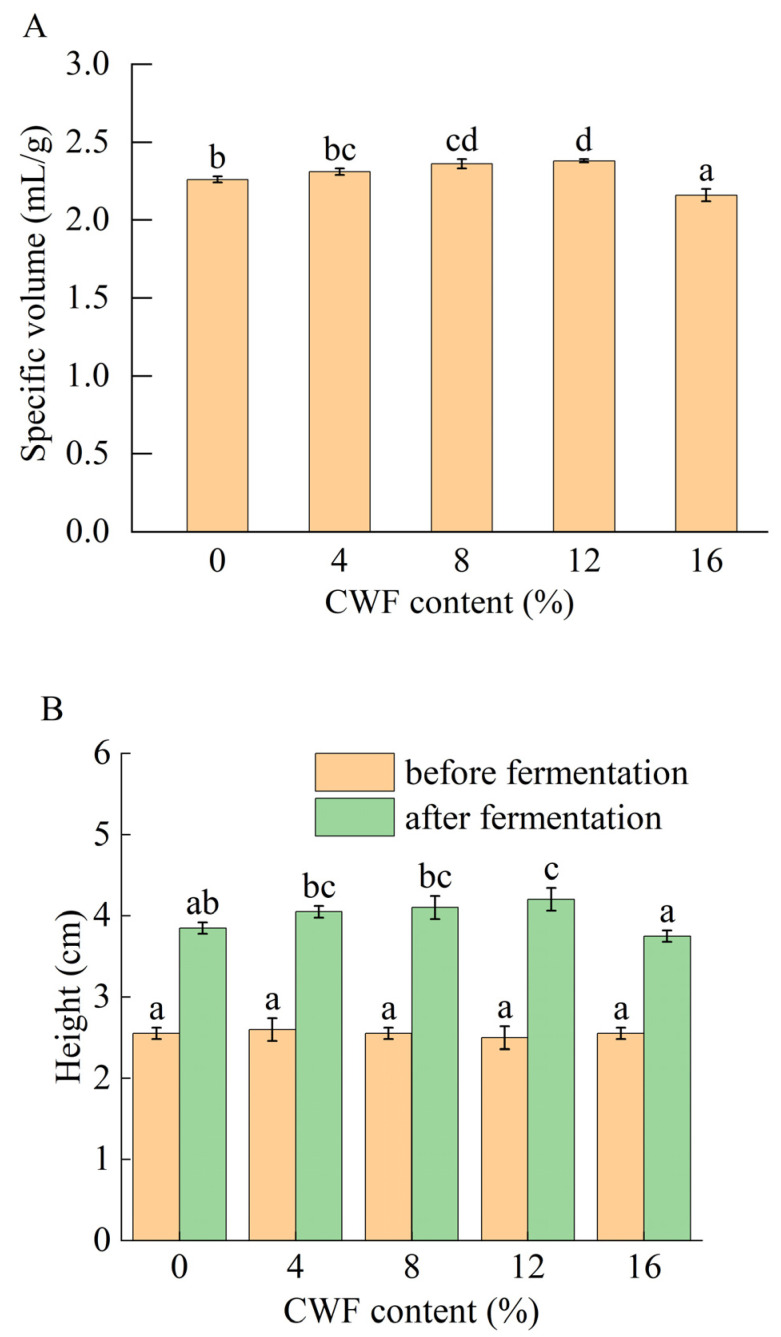
(**A**) Specific volume of steamed cake with 0%, 4%, 8%, 12%, and 16% carrot whole flour. (**B**) Height variation (before and after fermentation) of steamed cake with 0%, 4%, 8%, 12%, and 16% carrot whole flour. Different letters in the same filling pattern represented significant differences among different carrot additions (*p* < 0.05).

The specific volume had a strong influence on the decisions of consumers. Figure 1 illustrated the specific volume and height variations in steamed cakes with different CWF substitution levels. The specific volume of steamed cake initially increased, followed by a decrease. When the CWF addition was 12%, the specific volume of steamed cake significantly increased from 2.29 to 2.38 mL/g. ln addition, the rate of change in the height of the steamed cake increased from 50.98% to 68%. This might be because of covalent and non-covalent interactions between gluten and CPs, which positively affected the gluten network formation and consequently increased the specific volume of the steamed cake [33]. In addition, CWF had a higher reduced sugar content than wheat flour [34], and sugar was a major source of yeast fermentation, which promoted yeast reproduction. When the CWF addition was higher than 12%, the specific volume significantly decreased. During the steaming process, the high fiber content in CWF combined with water, which could limit water availability for starch-gluten network development. Consequently, CWF resulted in an underdeveloped gluten network and a reduction in steamed cake specific volume [35].

#### 3.2.2. Texture Analysis

Texture represented a critical quality parameter in product evaluation. An instrumental texture profile analysis (TPA) provided objective physical parameters that correlated with sensory perception, including hardness, springiness, cohesiveness, chewiness, and adhesiveness. Hardness, springiness, and chewiness are quality parameters usually associated with sensory evaluation. Among them, hardness denotes the maximum force value at first compression [36]. Springiness relates to the force with which a sample can be recovered after the first compression [37]. Chewiness quantifies the mastication energy required to transform steamed cake into a swallowable bolus [38].

The texture properties of steamed cake with or without CWF are shown in Table 2. As the CWF content increased (0–12%), the hardness and chewiness of the steamed cakes tended to decrease. There was a positive correlation between chewiness and hardness; the trend of chewiness of steamed cake was consistent with hardness. The results indicated that with the addition of CWF, the steamed cake formed a softer and chewier texture. Increasing CWF resulted in a significant decrease in springiness and cohesiveness. This could be attributed to the high dietary fiber content in CWF, which hindered the development of the gluten network and reduced the elasticity and cohesion of the steamed cake [39]. The textural properties of steamed cakes were predominantly governed by the concurrent processes of protein denaturation and starch gelatinization during thermal processing [40].

#### 3.2.3. Color

**Figure 2:**
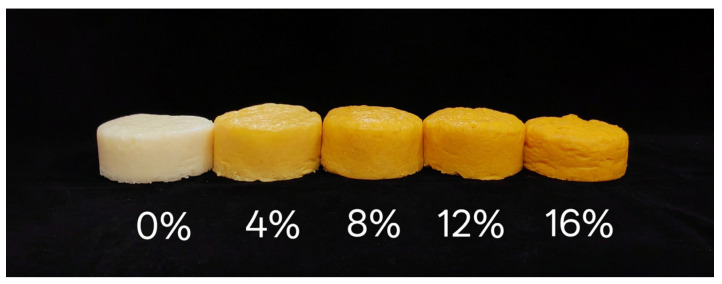
Color variation of steamed cake with 0%, 4%, 8%, 12%, and 16%.

The color of steamed bread influences consumer acceptability. With increasing CWF, the lightness (L*), redness (a*), yellowness (b*), and ΔE values of the steamed bread varied significantly (*p* < 0.05). It was obvious from Table 3 that the steamed bread L* decreased, whereas a* and b* increased with the addition of CWF, indicating that with increasing CWF, steamed breads were darker, reddish, and yellowish. The color variation of steamed cake with 0%, 4%, 8%, 12%, and 16% is presented in Figure 2. The color of starchy foods comes from the intrinsic color given by individual pigments and the color formed during the cooking process. This color change in the steamed cake might be due to carotenoids in the carrot. The ΔE value gradually increased, revealing that the overall color of the steamed cake was increasingly different from that of the control group due to the increase in the additional amount of CWF. The trend of color change (L*, a*, and b*) aligned with a study reported by Kowalczewski et al. [41].

#### 3.2.4. Sensory Evaluation

**Figure 3:**
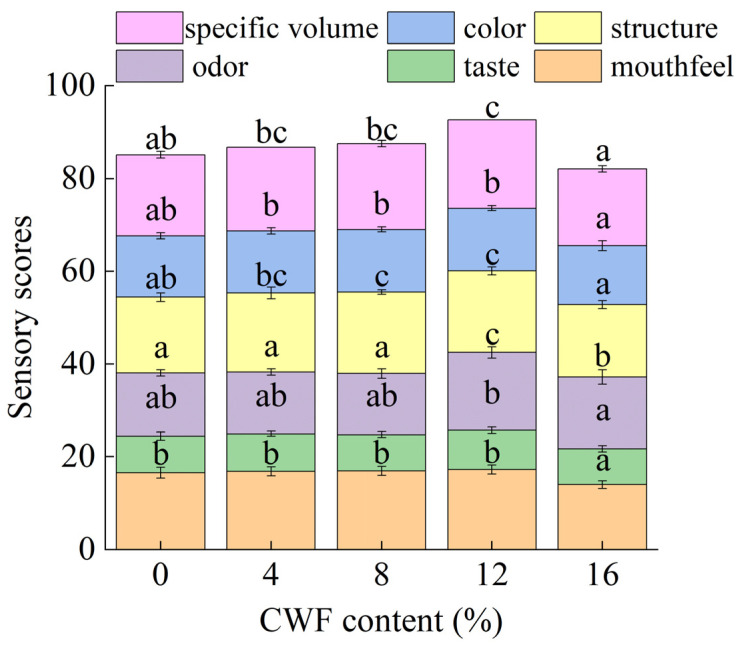
Sensory scores of steamed cake with 0%, 4%, 8%, 12%, and 16% carrot whole flour. Different letters in the same filling pattern denoted significant differences among different samples (*p* < 0.05).

The sensory characteristics of steamed cake were analyzed using radargram, and the specific volume, color, structure, odor, taste, and mouthfeel of steamed cake with different levels were scored comprehensively, and the results are shown in Figure 3 and Supplementary Material Table S2. When the CWF addition was 12%, the steamed cake had the largest area proportion in the radargram and the highest overall score. When the CWF addition was higher than 12%, the scores of various aspects decreased, which were manifested as rough texture, darker color, and other characteristics. The observed alteration in color aligned with the results of the color change in Section 3.2.3. Similarly, Wang et al. [42] reported that with increasing rose powder content, the steamed bread darkened, which led to lower acceptability scores. Excessive CWF led to the hardening of the steamed cake. Wu et al. [43] demonstrated that hardness in steamed bread exhibited a significant negative correlation with consumer acceptability. From the sensory analysis, the mouthfeel of the steamed cake increased and then decreased with the increase of CWF, which was consistent with the change in hardness in the texture analysis (Section 3.2.2). The adhesiveness showed a decreasing trend with higher CWF levels. This suggested that the incorporation of CWF may contribute to a low adhesive texture, potentially affecting the mouthfeel and sensory perception of the product. Based on the specific volume, texture, and sensory attributes of the steamed cake, the optimum dose of CWF in the steamed cake was 12%.

### 3.3. Gluten Properties

#### 3.3.1. Free Sulfhydryl and Disulfide Bond Content

Gluten consists of glutenin and gliadin. The covalent crosslinking of high molecular weight gluten subunits (HMW-GS) and low molecular weight gluten subunits (LMW-GS) through intermolecular disulfide bonds formed “head-to-tail” bonds that constituted the elastic backbone of gluten and created an amorphous three-dimensional network [44]. The free sulfhydryl content served as an indicator of gluten network stability, while disulfide bonds were essential for maintaining the structural integrity of the gluten network.

**Figure 4:**
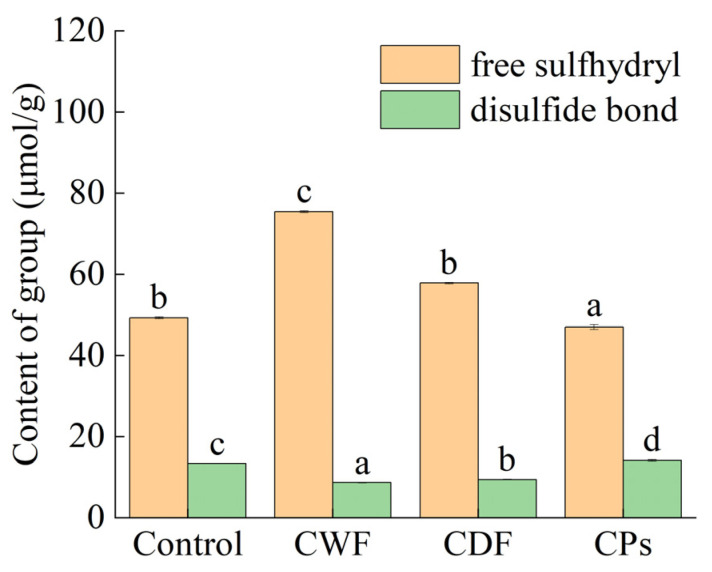
Effect of CWF, CDF, and CPs on the free sulfhydryl and disulfide bonds of gluten. CWF: carrot whole flour; CDF: carrot dietary fiber; CPs: carrot polyphenols. Different letters in the same filling pattern denote significant differences among different samples (*p* < 0.05).

The effect of CWF, CDF, and CPs on the free sulfhydryl and disulfide bond of gluten is shown in Figure 4. CDF increased the free sulfhydryl content and decreased the disulfide bond content, whereas CPs decreased the free sulfhydryl content and increased the disulfide bond content. CDF disrupted the development of the gluten network, and non-covalent interaction between CDF and gluten induces the formation of hydrogen bonds, thus interfering with the covalent cross-linking between gluten proteins [45]. Ding et al. [46] demonstrated that dietary fiber compromised the intermolecular interactions of gluten, resulting in the disruption of disulfide bonds.

However, it should be noted that CPs served as a source of antioxidants, promoting the reduction of disulfide bonds (SS) to free sulfhydryl groups (-SH). The results suggest that disulfide bond formation occurs primarily via intramolecular reactions, and this effect is strongly outweighed by the antioxidant properties of CPs. Jia et al. [47] reported esterification of phenolic acid-facilitated protein network formation via covalent and non-covalent cross-linking in gluten systems. Manu et al. [48] showed a significant positive correlation between disulfide bond content and wheat-based products hardness. CDF reduced the disulfide bond and caused a decrease in the hardness of the steamed cake.

#### 3.3.2. Intermolecular Interaction Force

Hydrogen bonds play a role in stabilizing the gluten network structure, and a hydrophobic interaction refers to the force formed on the hydrophobic side of the protein to avoid water molecules. Protein molecules build the gluten network structure through hydrogen bonds, hydrophobic interactions, and other forces, and the number of these chemical bonds and the ratio of each play a decisive role in the gluten network [49].

**Figure 5:**
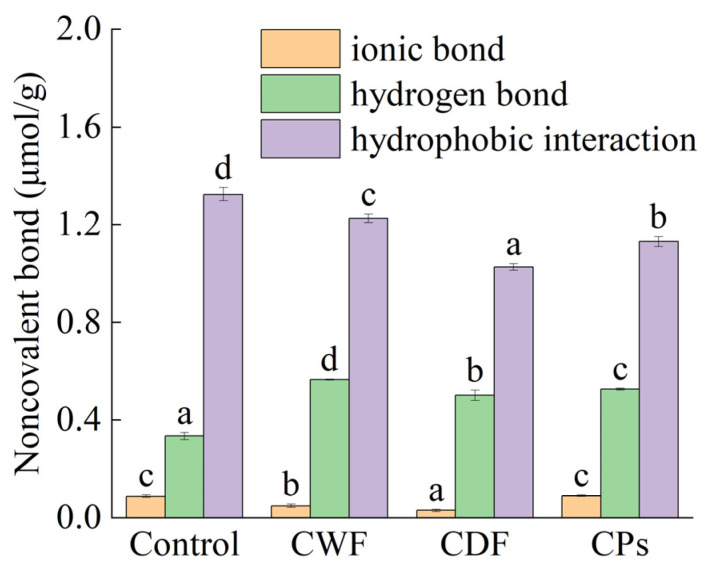
Effect of CWF, CDF, and CPs on the noncovalent bond of gluten. CWF: carrot whole flour; CDF: carrot dietary fiber; CPs: carrot polyphenols. Different letters on the same fill pattern denoted significant differences among different samples (*p* < 0.05).

The effect of CWF, CDF, and CPs on the intermolecular interaction of gluten is shown in Figure 5. It is shown that ionic bonds contribute little to the formation of wheat gluten [50]. Shewry et al. [51] showed that protein peptide chains were mainly maintained by hydrogen bonds, and when the hydrogen bond was exposed, the peptide chains were randomly arranged and aggregated with each other, and the hydrogen bond made the peptide chains form a stable structure. The hydrogen bond content of CWF, CDF, and CPs in gluten were higher than in the control. The increase in hydrogen bonds indicated that CWF significantly promoted hydrogen bond cross-linking between CPs and gluten molecules in the system. Qin et al. [52] reported that the addition of tea polyphenols increased the internal hydrogen bond of gluten. Nawrocka et al. [53] demonstrated that dietary fiber enhanced the hydrogen bond interactions of gluten. CWF reduced the hydrophobic interactions of gluten compared to the control. CDF and CPs also inhibited the hydrophobicity of gluten. In accordance with a study reported by Yang et al. [54], insoluble dietary fiber from apples exerted an inhibitory effect on hydrophobic interactions to strengthen the structure of the gluten network. CPs caused gluten to form more hydrogen bonds, which replaced hydrophobic bonds, resulting in fewer hydrophobic interactions [55]. In conclusion, CWF increased the hydrogen bond and decreased the hydrophobic interaction of gluten under the combined influence of CDF and CPs, and CPs contributed more than CDF. CPs stabilized the gluten structure by strengthening the hydrogen bond, causing an enhancement in the specific volume of steamed cake [56].

#### 3.3.3. Secondary Structure 

The secondary structure of proteins is intricately linked to the structure and strength of their networks. The region of amide I, ranging from 1700 to 1600 cm^−1^, was used to quantify the proportion of the protein’s secondary structure. The regions at 1600–1644 and 1685–1700 cm^−1^ correspond to the β-sheet, followed by 1652–1660 cm^−1^ to the α-helix, 1660–1685 cm^−1^ to the β-turn, and 1644–1652 cm^−1^ to the random coil [57,58].

**Figure 6:**
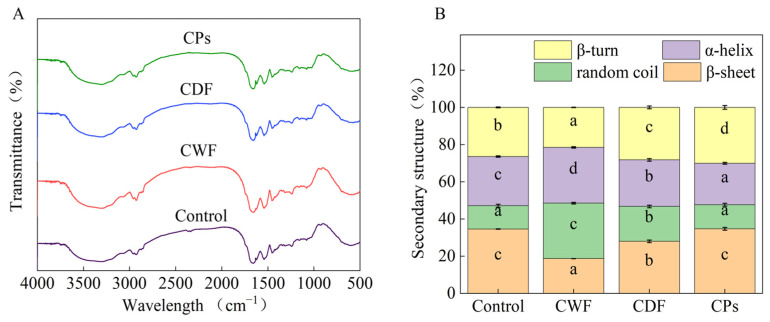
(**A**) Effect of CWF, CDF, and CPs on the FTIR spectra of gluten. (**B**) Effect of CWF, CDF, and CPs on the protein secondary structure. CWF: carrot whole flour; CDF: carrot dietary fiber; CPs: carrot polyphenols. Different letters in the same filling pattern denoted significant differences among different samples (*p* < 0.05).

The effect of CWF, CDF, and CPs on the gluten secondary structure is shown in Figure 6. It has been demonstrated that protein stability is correlated with the β-sheet content, and the random coil is thought to be a disordered structure [59,60]. CWF, CDF, and CPs decreased β-sheet content, and CWF, CDF, and CPs increased random coil content. CWF interfered with the development of gluten, leading to a shift from a stable protein structure to a more unstable one. The wheat product was fortified with various components such as dietary fiber, polyphenols, polysaccharides, and other bioactive phytochemicals [61]. Previous studies had shown that these components could have a direct or indirect impact on the secondary structure of gluten [62]. Gluten competed with CDF for water absorption, diminishing the water utilization efficiency of gluten during the formation process, promoting gluten aggregation [63,64]. The reduction in β-sheet content was correlated with the degree of protein aggregation [65]. Chlorogenic acid (CA) is the major phenolic acid in carrots. Zhang et al. [66] reported that CA caused glutenin to change from an organized structure to a disorganized structure. CA was placed inside the hydrophobic space of gliadin, which elongated the structure of gliadin and made the arrangement more disordered. As shown in Figure 6B, we can conclude that CDF and CPs together influenced the secondary structure of gluten, but CDF exerted a greater influence on the secondary structure of gluten than CPs. CDF resulted in a reduction in β-sheet content, which subsequently influenced the quality characteristics of steamed cake and led to a decrease in steamed cake hardness [67]. Results of the secondary structure confirmed the consistency of previous covalent interaction results (Section 3.3.1). CWF disrupted the disulfide bonds and unfolded the gluten network, resulting in fewer extended chains, which led to a decrease in β-sheet content [68]. 

### 3.4. Starch Properties

#### 3.4.1. Pasting Properties

**Figure 7:**
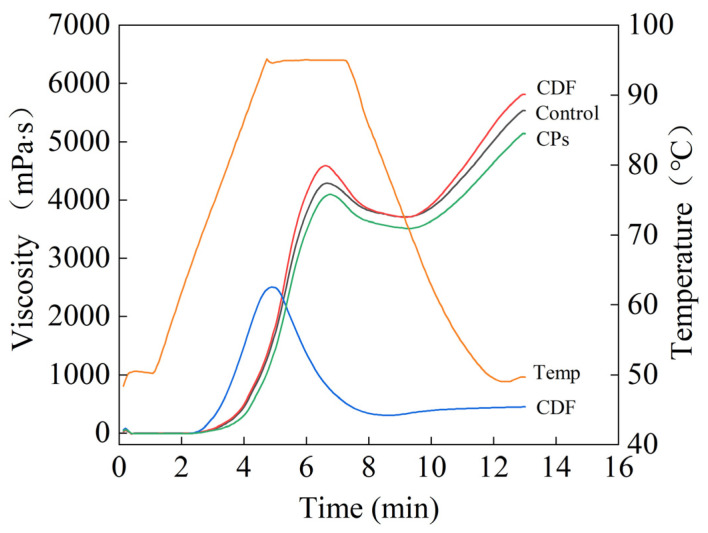
Effect of CWF, CDF, and CPs on the pasting profiles of starch. CWF: carrot whole flour; CDF: carrot dietary fiber; CPs: carrot polyphenols.

The pasting characteristics of starch are closely linked to the quality of starchy foods and are a crucial parameter for assessing the cooking quality of starchy foods. Figure 7 represents the pasting profiles of starch. Table 4 presents the related pasting parameters of the samples. The peak viscosity and final viscosity of CPs and CDF starch were less than CK. CWF starch exhibited a significantly higher peak and final viscosity than the control. The hydrophilic groups in CDF and CPs competed with starch for water absorption in the mixed system, leading to a reduction in starch viscosity. The viscosity of CWF starch was not affected by CDF and CPs and might be dominated by other components. Chen et al. [69] observed that pectin can increase the viscosity of starch. CWF contained pectin [70], which might enhance the viscosity of CWF starch.

The breakdown indicated the structural integrity of starch granules during the pasting process; the lower the breakdown, the more stable the starch granules [71]. CDF significantly resulted in an increase in the breakdown value, and CPs slightly increased the breakdown value compared with the control. The research revealed that CDF exerted a greater impact on the breakdown value than CPs, suggesting that CDF had a greater role in the breakdown value of CWF starch.

The setback value is an index to measure the short-term retrogradation of the starch system. CWF increased the setback value compared to the control. Starch systems with CDF and CPs showed lower setback values, which were similar to the results of Li et al. [72]. CDF and CPs acted as a physical barrier among the starch chains, and they hindered the arrangement of starch chains during cooling. CDF and CPs had no influence on the setback value of CWF starch, and might be dominated by other components.

The thermal stability of starch was affected by the increase or decrease in pasting temperature [73]. CDF decreased the pasting temperature of starch, indicating that CDF disrupted the thermal stability of starch, while CPs increased the pasting temperature of starch, which increased the thermal stability of wheat starch.

In conclusion, in terms of pasting characteristics, the contribution of CDF was greater than that of CPs, and CDF destabilized the starch granules, which had a negative effect on the stability of the wheat product.

#### 3.4.2. Short-Range Ordered Analysis

**Figure 8:**
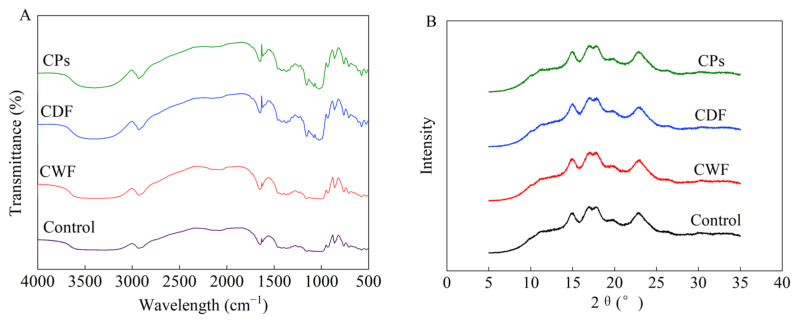
Effect of CWF, CDF, and CPs on the FTIR spectra of starch. (**A**) Effect of CWF, CDF, and CPs on the XRD spectra of starch (**B**). CWF: carrot whole flour; CDF: carrot dietary fiber; CPs: carrot polyphenols.

The FT-IR spectra of samples are presented in Figure 8A. All samples had similar characteristic peaks at 3600–3000 cm^−1^. This characteristic peak usually indicated the formation of O-H bonds between and within molecules [74]. The absorption peaks near 2930 cm^−1^ and 1647 cm^−1^ are characteristic of the antisymmetric stretching vibration of CH_2_ and aromatic C=C stretching, respectively [75,76]. The observed spectral alterations imply that the formation of CWF-starch complexes potentially entails synergistic interactions modulating both hydrogen-bonding architectures and bioactive polysaccharide constituents.

The absorption peak at 1047 cm^−1^ corresponded to the crystalline region, the absorption peak at 1022 cm^−1^ corresponded to the amorphous region, and the degree of starch order was reflected by the ratio of the intensities of the two peaks [77]. The starch’s organized structure was also related to the value of R995/1022, which indicated that some degree of double helix formation occurred [78]. As shown in Table 5, CWF, CDF, and CPs reduced the short-range order of starch. CDF decreased the width of the absorption peak at 3600–3000 cm^−1^ of starch, indicating hydrogen bond breaking [79]. The hydroxyl structure of CDF cannot be well aggregated with free starch chains through the hydrogen bond, disrupting the double helix structure of starch [80]. Liang et al. [81] revealed that the incorporation of insoluble dietary fiber decreased the R1047/1022 values of the gel system, indicating that the incorporation of insoluble dietary fiber decreased the stability of the gel structure. CPs decreased the R1047/1022 and R995/1022 ratio, which suggests that CPs inhibited the formation of short-chain self-assembly and stabilization of helical conformations [82]. Similarly, Li et al. [83] reported that CA interacted with starch and disrupted the ordered structure of starch. In summary, CDF and CPs had a negative effect on starch structure, characterized by a decrease in short-range order. CDF and CPs disrupted the short-range order of starch and its thermal stability, bringing about a reduction in steamed cake springiness and cohesiveness, respectively [84].

#### 3.4.3. X-Ray Diffraction

The interior of the starch granule consisted of crystalline and amorphous regions and was usually considered a semi-crystalline or partially crystalline polymer. The crystalline nature of starch gives it a distinctive X-ray diffraction pattern, which is generally classified as A-type, B-type, and C-type [85]. Figure 8B presents the XRD patterns for the starch samples.

All the starches had characteristic diffraction peaks at approximately 2θ of 15, 17, 18, and 23°, which suggests that these starches all belong to the A-type crystal structure [86]. Another prominent peak was approximately located at a 2θ value of 20° and identified as V-type crystallinity [87]. CWF-, CDF-, and CP-fortified starches exhibited higher peak intensities at 2θ of 14.8, 16.9, 17.8, and 19.8° compared to CK, indicating that CWF facilitated the recrystallization of starch [30]. Relative crystallinity results are presented in Table 5. CWF starch, CDF starch, and CP starch had higher crystallinity than CK. The competition between the hydrophilic groups of CDF and water molecules leads to weakened binding between starch and water and increased interactions between starch chains [88]. Liu et al. [89] showed that insoluble dietary fiber from rice bran altered the diffraction peak values of the crystalline structure of starch and significantly increased the relative crystallinity. CPs could bind to the starch chains and cause the rearrangement of the starch chains [90]. As shown in Table 5, CDF and CPs together influenced starch crystallinity. The addition of CWF increased the crystallinity of the starch, indicating that the presence of CWF made the steamed cake susceptible to staling. CDF and CPs caused an elevation in crystallinity, leading to a loss of steamed cake springiness [91].

## 4. Conclusions

This study aimed to analyze the effect of carrot flour on the quality of steamed cake. The results showed that the quality of the steamed cake was better at an additional level of 12%. The interaction between CWF and protein–starch is crucial for making carrot steamed cake. The effects of CWF and its active ingredients (CDF and CPs) on gluten properties and starch properties were studied. In terms of gluten properties, CWF induced a 35.16% reduction in the disulfide bond and converted the β-sheet to a random coil mainly due to CDF, concomitantly lowering the hardness of steamed cake by 13.3%. CWF caused gluten to form more hydrogen bonds (from 0.335 to 0.566 μmol/g) (*p* < 0.05), consequently increasing the specific volume (from 2.29 to 2.38 mL/g) (*p* < 0.05). Moreover, CPs contributed more than CDF. In terms of starch properties, due to the combined effects of CDF and CPs, CWF reduced the starch short-range order by 6.13% and increased the breakdown value by 53%, decreasing the elasticity and cohesiveness. CDF and CPs caused starch molecules to rearrange, leading to an increase in crystallinity by 6.2% and reducing the springiness of the steamed cake. In the future, we can mitigate the adverse effects of CWF on gluten and starch by using enzymes to obtain wheat carrot products that combine high taste quality and health. This study establishes a theoretical foundation for the development of functional wheat-carrot-based products and contributes to dietary intervention strategies for chronic disease prevention.

## Figures and Tables

**Table 1:** Chemical compositions of raw materials.

	Wheat Flour	Carrot Whole Flour
Moisture (g/100 g)	12.51 ± 0.03 b	7.45 ± 0.07 a
Protein (g/100 g)	12.10 ± 0.23 b	6.17 ± 0.21 a
Fat (g/100 g)	0.86 ± 0.08 a	2.27 ± 0.15 b
Ash (g/100 g)	0.40 ± 0.01 a	5.57 ± 0.06 b
Total dietary fiber (g/100 g)	1.09 ± 0.10 a	18.52 ± 0.14 b
Total polyphenolic (mg/100 g)	75.93 ± 0.85 a	190.58 ± 0.59 b
Carotenoid (mg/100 g)	0.49 ± 0.03 a	135.35 ± 1.93 b

Note: Values were calculated on a dry basis. Different letters represented the distinctions between diverse materials (*p* < 0.05).

**Table 2:** Textural properties of steamed cakes with different levels of carrot whole flour.

Samples	Hardness (g)	Springiness	Cohesiveness	Chewiness (g)	Adhesiveness
Control	7.50 ± 0.28 ^b^	9.34 ± 0.15 ^d^	0.72 ± 0.03 ^d^	43.78 ± 1.83 ^d^	5.23 ± 0.12 ^d^
CWF4	7.38 ± 0.18 ^b^	8.86 ± 0.08 ^c^	0.65 ± 0.02 ^c^	42.64 ± 1.06 ^d^	4.81 ± 0.08 ^b^
CWF8	7.25 ± 0.12 ^b^	8.59 ± 0.08 ^b^	0.61 ± 0.01 ^b^	37.91 ± 1.19 ^b^	3.97 ± 0.09 ^a^
CWF12	6.50 ± 0.16 ^a^	8.45 ± 0.09 ^b^	0.61 ± 0.02 ^b^	32.14 ± 1.18 ^a^	3.91 ± 0.14 ^a^
CWF16	8.75 ± 0.10 ^a^	8.01 ± 0.18 ^a^	0.57 ± 0.02 ^a^	40.23 ± 0.97 ^c^	5.02 ± 0.14 ^c^

Note: CWF4, CWF8, CWF12, and CWF16 represent the steamed cakes with 4%, 8%, 12%, and 16%, respectively. Different letters within a column denote significant differences (*p* < 0.05).

**Table 3:** Effect of carrot whole flour on color attributes of steamed cake.

Samples	L*	a*	b*	ΔE
Control	77.64 ± 0.21 ^e^	0.41 ± 0.04 ^a^	14.21 ± 0.04 ^a^	
CWF4	65.59 ± 0.76 ^d^	11.40 ± 0.60 ^b^	40.36 ± 0.38 ^b^	31.20 ± 0.27 ^a^
CWF8	63.96 ± 0.35 ^c^	17.10 ± 0.26 ^c^	48.64 ± 0.59 ^c^	41.03 ± 0.50 ^b^
CWF12	60.85 ± 0.33 ^b^	18.14 ± 0.17 ^d^	53.34 ± 0.33 ^d^	46.49 ± 0.20 ^c^
CWF16	58.76 ± 0.17 ^a^	20.39 ± 0.23 ^e^	55.80 ± 0.38 ^e^	50.24 ± 0.38 ^d^

Note: CWF4, CWF8, CWF12, and CWF16 represent steamed cakes with 4%, 8%, 12%, and 16%, respectively. Different letters within a column denote significant differences (*p* < 0.05).

**Table 4:** Effect of CWF, CDF, and CPs on the pasting characteristics of starch.

Samples	PV (mPa·s)	BD (mPa·s)	FV (mPa·s)	SB (mPa·s)	PT (°C)
Control	4287.50 ± 28.99 ^c^	583.00 ± 50.91 ^a^	5532.50 ± 14.85 ^c^	1878.00 ± 24.04 ^c^	77.85 ± 0.57 ^b^
CWF	4593.50 ± 26.16 ^d^	892.00 ± 59.40 ^b^	5814.00 ± 5.66 ^d^	2062.50 ± 9.19 ^d^	77.50 ± 0.07 ^b^
CDF	2507.00 ± 8.49 ^a^	2206.00 ± 21.21 ^c^	448.00 ± 9.90 ^a^	147.00 ± 22.63 ^a^	69.43 ± 0.04 ^a^
CPs	4098.50 ± 33.23 ^b^	610.50 ± 10.61 ^a^	5140.00 ± 26.87 ^b^	1652.00 ± 16.97 ^b^	81.45 ± 0.00 ^c^

Note: PV: peak viscosity; FV: final viscosity; BD: breakdown viscosity; SB: setback viscosity; and PT: pasting temperature. CWF: carrot whole flour; CDF: carrot dietary fiber; CPs: carrot polyphenols. Distinct letters within a column denote significant differences (*p* < 0.05).

**Table 5:** Effect of CWF, CDF, and CPs on the relative crystallinity and IR ratio of starch.

Samples	R1047/1022	R995/1022	Relative Crystallinity
Control	0.978 ± 0.017 ^c^	0.989 ± 0.010 ^c^	29.64 ± 0.38 ^a^
CWF	0.918 ± 0.008 ^b^	0.948 ± 0.006 ^b^	31.48 ± 0.06 ^b^
CDF	0.895 ± 0.009 ^ab^	0.892 ± 0.002 ^a^	32.29 ± 0.97 ^b^
CPs	0.880 ± 0.002 ^a^	0.898 ± 0.008 ^a^	32.72 ± 0.79 ^b^

Note: CWF: carrot whole flour; CDF: carrot dietary fiber; CPs: carrot polyphenols. Distinct letters within a column denote significant differences (*p* < 0.05).

## Data Availability

The raw data supporting the conclusions of this article will be made available by the authors on request.

## Supplementary Materials

The following supporting information can be downloaded at: https://www.mdpi.com/article/10.3390/foods14111964/s1, Table S1: Sensory evaluation standard of steamed cake, Table S2: Sensory properties of steamed cakes with different levels of carrot whole flour.
