# Supplementary material for: From Molecular to Macroscopic: Dual-Pathway Regulation of Carrot Whole Flour on the Gluten-Starch System
Source: Foods. 2025 May 31;14(11):1964. doi: 10.3390/foods14111964 (PMC12155530; doi:10.3390/foods14111964)
Supplement: Supplementary file 1 [file foods-14-01964-s001.zip › foods-3637293-supplementary.pdf]

Supplementary Materials

Table S1. Sensory evaluation standard of steamed cake

Indicator	Scoring Criteria	Score
Specific Volume	Specific volume of 2.5mL-g-1 is set as full marks, 1 mark is deducted for each 0.1mL-g-1 less	20
Color	Uniform color distribution	10-15
	Basically uniform color distribution	5-10
	Non-uniform color distribution	0-5
Structure	Uniform air pore distribution	16-20
	Basically uniform air pore distribution	12-16
	Non-uniform air pore distribution	8-12
Odor	Fermented odor and strong smell	10-15
	Fermented odor and light smell	5-10
	Impure odor or severe sourness	0-5
Taste	special taste from fermentation	8-10
	Basically pure taste from fermentation	5-7
	Non-special taste or bitterness from fermentation	0-5
Mouthfeel	Suitable softness and hardness, flexible	16-20
	Slight hardness or softness, slight flexible	12-16
	excessive softness or hardness, non-flexible	8-12

Table S2. Sensory properties of steamed cakes with different levels of carrot whole flour

	Properties / Samples	Control	CWF4	CWF8	CWF12	CWF16
Sensory	Specific Volume	17.50±0.71ab	18.00±0.00bc	18.50±0.71bc	19.00±0.00c	16.50±0.71a
	Color	13.20±0.63ab	13.40±0.70b	13.50±0.53b	13.50±0.53b	12.70±1.06a
	Structure	16.30±0.95ab	17.00±1.25bc	17.50±0.53c	17.60±0.84c	15.60±0.84a
	Odor	13.60±0.68a	13.30±0.68a	13.20±1.03a	16.80±1.23c	15.50±1.58b
	Taste	7.90±0.63ab	8.10±0.88ab	7.80±0.57ab	8.40±0.70b	7.70±0.68a
	Mouthfeel	16.60±1.17b	16.90±0.99b	17.00±0.94b	17.30±0.95b	14.00±0.82a
	Total score	84.60±1.52b	86.50±1.35c	90.50±1.57c	92.60±1.35d	82.00±1.65a

Note: CWF4, CWF8, CWF12 and CWF16 represented the steamed cake with 4%, 8%, 12% and 16%, respectively. Different letters in the same row indicated significant differences.
